# Expand the scorecard for health-care reform to achieve a better result and enhance clinical and translational science

**DOI:** 10.1017/cts.2018.333

**Published:** 2018-12-07

**Authors:** Barry S. Coller

**Affiliations:** Rockefeller University, 1230 York Avenue, New York, NY, USA

**Keywords:** Health-care reform, Affordable Care Act, clinical and translational science, medical education, resource allocation

## Abstract

Clinical and translational science is vitally dependent on the nation’s underlying health-care policies and programs. In a reciprocal fashion, data generated by clinical and translational research can inform both health policy and health-care delivery. It is important, therefore, to rate health reform proposals comprehensively on a set of criteria that reflect the broad goals of reform, including the potential impact on clinical and translational science and medical education. I propose that the criteria include achieving universal coverage, reducing administrative costs, retaining one’s chosen primary care physician, encouraging care coordination, empowering physicians, freeing industry from choosing and administering health plans, providing choice of specialists and hospitals, providing patient education, preventing patient overuse of services, rationalizing resource allocation, encouraging competition, limiting government’s role, supporting medical education, training, and research, and freeing industry to make personnel decisions based on business criteria rather than the impact on health-care costs to the company. I discuss the rationale for each element and offer a rating of current proposals relative to a proposal previously made.

Clinical and translational science is vitally dependent on the nation’s underlying health-care policies and programs. In a reciprocal fashion, data generated by clinical and translation research can inform both health policy and health-care delivery programs, with the growing body of implementation science data especially valuable in optimizing the effectiveness of new innovations. Thus, the clinical and translational science community has a vital interest in the intense national focus on health-care reform. It is important, therefore, to rate proposals comprehensively on a set of criteria that reflect the broad goals of reform, including the potential impact on clinical and translational science and medical education.

With the intense focus on the future of the Affordable Care Act and the alternative plans proposed by Congressional Republicans and President Trump, it is important to rate proposals comprehensively since the structures of the plans and the incentives they create will have profound effects on the quality of health care, medical education, and clinical and translational science, as well as the nation’s economic stability. Despite the intense partisan divides, there are a number of criteria one can use to assess a plan that are rooted in consensus about desirable features in health-care delivery. This study offers a scorecard to assess the plans based on such criteria, but excluding the relative financial contributions by different sources since there is no broad consensus on that issue.

1. Does the reform provide universal health care? If not, what percentage of the population will have coverage, and will coverage vary by age, wealth, job status, or preexisting medical conditions?

Why? Universal coverage is a moral obligation, simplifies many administrative and legal aspects, and prevents distortions in economic decision-making based on the impact of the decision on medical coverage. It is especially important for clinical and translational science since it has major implications for access to health care, health disparities based on restricted access, and the pool of individuals who can participate in research studies requiring clinical care.

2. Does the reform reduce or eliminate administrative costs that do not contribute meaningfully to health care?

Why? The United States has the highest percentage of administrative costs among the 11 nations analyzed by the Commonwealth Fund [1], and the US doctors spend the most time on both payment and treatment-approval insurance paperwork [1]. Potential savings may be more than US$500 billion per year [2]. The health-care economist, Uwe Reinhardt told *Managed Care* magazine in 2013, “Our hospitals spend twice as much on administration as any hospital anywhere in the world […].” He noted that if the nation cut the cost of health-care administration in half, the savings would be enough to insure everyone. Reducing health-care costs would also provide savings in the discretionary component of federal and state budgets that could be allocated to medical research.

3. Does the reform encourage stable relationships between patients and their primary care physicians?

Why? A stable, long-term relationship with a trusted primary care physician results in more efficient and higher quality care, with greater emphasis on prevention and health promotion. Moreover, the trust built up over time between patient and physician is also likely to enhance patient acceptance of important new preventive strategies and novel therapies. Switching primary care physicians is not only expensive and inefficient but also poses medical risks.

4. Does the reform reduce patients’ health insurance paperwork?

Why? The Commonwealth Fund study also found that the US patients also spend the most time on health insurance paperwork, a hidden cost that also translates into frustration and discontent.

5. Does the reform encourage the coordination of care by medical specialists, hospitals, and long-term health facilities, especially for patients with complex, chronic diseases, which are most costly?

Why? Current estimates indicate that 5% of the patients with the greatest medical needs account for 50% of the total health-care costs, making the care of this population the greatest opportunity to reduce costs. Removing the constraints on patient care delivery that are implicitly imposed by current reimbursement policies should also provide greater opportunity for research on optimizing the care of such patients.

6. Does the reform empower physicians and nurses by providing them with the freedom they need to use their creativity to address their patients’ health needs?

Why? Since the fee-for-service system has the potential for abuse if providers perform unneeded services, an elaborate apparatus to prevent such abuse has developed that diverts the efforts of dedicated professionals from patient care to detailed documentation and obtaining prior approvals for needed services. Shifting to quality-based or value-based physician payments seems like a reasonable alternative, but the metrics chosen inevitably distort professional judgment and behavior as the measured metrics become overemphasized [3]. As noted in question number 5, the greater freedom to allocate resources should translate into new opportunities for research, especially in implementation science.

7. Does the reform free companies from having to decide on a yearly basis how to provide health care coverage to their employees?

Why? Currently companies devote considerable resources each year to selecting insurance plans for their employees, including hiring consultants. This is a hidden cost of our health-care system.

8. Does the reform allow individuals to select the hospital and specialists they prefer?

Why? Many health plans require that patients choose from the insurer’s roster of specialists and hospitals or face incurring significant charges. Often it is difficult to know exactly which specialists and hospitals are on the roster at the time of plan selection, and rosters change over time, potentially interrupting a relationship between patient and specialist. This leads to inefficiencies in transferring the patient’s care and increases the potential for inadvertent medical errors.

9. Does the reform educate patients about measures they should take to maintain their health and the costs of medical care?

Why? Patient education is at the core of disease prevention, and it is important for patients to have information about the costs of the services they require so that they have a realistic idea of the economics of health care and can participate more knowledgably in public discussions about health-care policies and payments. This also provides opportunities to study the optimal methods for patient education as an important component of implementation science. If established as two-way communication, it would allow for greater engagement by the patient community in all phases of clinical and translational research.

10. Does the reform include incentives to reduce patient requests for unneeded or futile medical care?

Why? It requires delicacy to balance the goal of insuring that everyone who needs medical care has access to that care while preventing abuses of the system in which patients request unneeded or futile care. Rigorous, empiric research on balancing these goals is an extremely high priority for optimizing the value of health-care programs.

11. Does the reform maximize the likelihood that decisions regarding allocation of resources are made based on total health-care costs and benefits?

Why? When decisions are made based on the economics of just a sliver of the total health-care system, it is unlikely that they will sum to the most rational allocation of total resources. For example, a hospital pharmacist may feel pressure to resist the introduction of a costly medication that will unbalance the pharmacy’s budget, even if the medication reduces total health-care costs by preventing future hospitalizations. Rigorous, empiric research is also needed to better understand the impact of the health-care reimbursement system on rational allocation of resources.

12. Does the reform encourage healthy competition among plans?

Why? Competition is a central tenet of our economic system because at its best it drives quality and efficiency. Single-payer systems lose the benefits that come from healthy competition and government-supported single-payer systems inevitably develop intrusive, inefficient, and demoralizing oversight mechanisms and attempt to manipulate provider behavior [2].

13. Does the reform limit government action to setting national minimum standards for health plan coverage, collecting and distributing payments to health systems, and setting national targets for total health care spending?

Why? Government at its best can set national standards for what should be included in a comprehensive health-care package and can collect and disburse funds efficiently. Government is least successful and most intrusive, however, when in the name of preventing fraud and abuse it micromanages or delays treatment.

14. Does the reform strengthen medical education, medical training, and medical research?

Why? The future of American medicine depends on the quality of medical student education, resident training, and biomedical research. Reforms that weaken academic medical centers and teaching hospitals will ultimately weaken medical care for all and stifle the clinical and translational research that is needed to continually improve health care.

15. Does the reform free industry to make personnel decisions based on business criteria rather than the impact on health-care costs to the company?

Why? When considering whether to add new employees, industry currently must consider the economic impact on its health-care costs in addition to the person’s potential contributions to the business and the cost of the person’s salary, especially if the company’s health-care benefits are partially or fully self-insured. This distorts optimal economic decision-making. For example, companies may encourage early retirement to decrease their exposure to the health-care costs of elderly employees even when the employees are the most skilled.

Table 1 provides subjective ratings of how the fee-for-service and single-payer models rank on each of the above criteria, along with the Affordable Care Act provisions and the Republican proposals. While they vary considerably in addressing the scorecard criteria, with the single-payer and Affordable Care Act ranking higher than the other 2, none uniformly ranks high. A universal health care reform program previously proposed is specifically designed to better align incentives so as to meet the goals indicated earlier [4]. The key elements in the proposal include:

1. Creating competing nonprofit organizations that comprise specialist physicians and hospitals, each with an equal share of governance that would offer plans to individuals on a yearly basis. These organizations would be paid a global fee each year by the government for the care of the patients who choose their plan (“capitation”), with appropriate adjustments for patient age, sex, health status, and poverty level, as well as regional costs of living. Additional positive adjustments would be made for hospitals engaged in research and education. This method of payment would free both hospitals and specialists from most of the costs of billing and collecting. In addition, it would create an incentive to avoid overuse of medical technology. Although a global fee can raise concerns about the incentive for underuse of medical services and technology to reduce costs, the increasing public availability of data on the quality of medical care provided by each organization would protect against inappropriate underuse of resources. Most importantly, this form of payment would empower physicians, nurses, and hospitals to creatively integrate the care of the patients with the most complex, and thus most expensive, disorders. The capitation method of payment also allows the government to better control total health-care cost. For example, it could link such costs to a defined percentage of the gross domestic product.
2. De-linking the individual’s choice of primary care physicians from their choice of specialist–hospital organization, allows them to retain a stable relationship with their primary care physicians even if they change their specialist physician–hospital organization.

Together, these 2 elements can achieve universal coverage, lower administrative costs, preserve competition among plans, eliminate the need for employers to select plans, encourage long-term relationships between patients and primary care physicians, and empower physicians, nurses, and hospitals to integrate care to maximize both quality and value. It would also free industry from having to research and offer plans each year and further free industry from having to factor employees’ likely health-care costs into their hiring and retention decisions. Collectively, these changes would also free the clinical and translational research enterprise from the strictures imposed by the current reimbursement policy noted earlier.

3. Providing information to patients to educate them about their health and the costs of their care. Every month, each individual would receive a communication geared to his or her age and sex indicating the preventive health measures they should consider (e.g., vaccinations and screening for colorectal cancer); the total amount and sources of money contributed for their health care (from them, their employers, state or federal government); and an itemized list and total cost of the health-care services they utilized.
4. Permitting individuals who have a positive balance of total health-care payments relative to total health-care costs at the time of their death to direct that the positive balance be given to a nonprofit organization such as their local church, or a governmental agency such as the National Institutes of Health.

**Table 1:** Health-care reform scorecard

	Pre-Affordable Care Act fee-for-service	Single payer	Affordable Care Act	Republican bills
Achieves universal coverage	–	+	±	–
Reduces administrative costs	–	+	±	–
Encourages stable relationship between patients and their primary care physician	–	+	±	–
Reduces patient’s health insurance paperwork	–	±	–	–
Encourages care coordination	–	–	±	–
Empowers physicians	–	–	±	–
Frees industry from choosing and administering health plans	–	+	–	–
Provides choice of specialists and hospitals	±	+	±	±
Provides patient education on their health and health-care costs	–	–	–	–
Discourages patient overuse of services	–	–	–	–
Rationalizes resource allocation	–	–	±	–
Encourage competition	+	–	+	+
Limits government’s role to setting standards and collecting/distributing funds	–	–	–	–
Supports medical education, training, and research	±	–	±	±
Frees industry to make personnel decisions based on business criteria rather than the impact on health-care costs to the company	–	+	–	–

The last 2 provisions would educate the public about both their health care and its costs, and would provide a modest incentive to avoid unneeded or futile medical care. They would also make the general public more knowledgeable as participants in the national debate about the fairest way to apportion the funding of health care from recipients, employers, and federal and state governments. In addition, integrating individualized public education into the health-care plan would provide an excellent research platform for optimizing that education. Expanding that interaction so that it is bi-directional would facilitate engagement of the patient community in all phases of clinical and translational research.

The author realizes that there are potentially many other ways than the ones proposed to score high on the above health-care reform scorecard and support medical education and clinical and translational science. In addition, the scorecard itself can certainly be refined and improved. The key issue is to recognize the importance of expanding public discussion of health-care reform beyond the narrow aspects that have dominated the headlines because there is much more to consider. Health-care reform is the country’s most important domestic challenge, with profound implications for the nation’s health, economic competitiveness, leadership role in medical education and clinical and translational science, and national security. It requires creativity, compassion, and commitments to social justice, medical research, and fiscal responsibility. It is essential to get it right.
